# Method shifting from long to short term contraceptives and its associated factors among reproductive age women, northwest Ethiopia

**DOI:** 10.1186/s40834-022-00207-7

**Published:** 2023-02-06

**Authors:** Niguse Desalegn, Melaku Kindie Yenit, Yohannes Ayanaw Habitu

**Affiliations:** 1 Metema Hospital, West Gondar zone, Gondar, Ethiopia; 2 Department of Epidemiology and Biostatistics, Institute of Public Health, University of Gondar, Gondar, Ethiopia; 3 Department of Reproductive and Child Health, Institute of Public Health, University of Gondar, Gondar, Ethiopia

**Keywords:** Method shifting, Contraceptives, Associated factors, Reproductive age

## Abstract

**Background:**

Even if long term contraceptives are more effective, efficient and tolerable choices, method shifting from long to short term contraceptives continued as a global challenge including Ethiopia. There is limited evidence on the proportion and factors associated with method shifting from long term to short term contraceptives in the country, specifically in the study area. Therefore, this study assessed the proportion and associated factors of method shifting from long term to short term contraceptives in Gondar city administration, northwest Ethiopia.

**Methods:**

Institution based cross-sectional study was conducted from February to June 2018 among reproductive age women who were long term contraceptive users. A total of 407 women of reproductive age were selected using systematic random sampling technique. Data were entered through Epi Info version 3.5.3 and analyzed using SPSS version 20. Bivariable and multivariable logistic regression analyses were employed to investigate factors associated with method shifting. Adjusted Odds Ratio with the corresponding 95% confidence intervals were used to show the presence and strength of association. Variables with *P*-value of < 0.05 in the multivariable model were considered to have statistically significant association with method shifting.

**Results:**

The overall proportion of method shifting from long to short term contraceptives was 48.5% [CI: 43.8, 53.3]. Having secondary level educational status [AOR = 0.18, CI = 0.07, 0.51], using long acting contraceptives for limiting purposes [AOR = 0.26, CI = 0.11, 0.60], and having enough counseling on long acting contraceptives during ANC visits [AOR = 0.20, CI = 0.08, 0.50] were factors negatively associated with method shifting, while receiving information about long acting contraceptives from colleague [AOR = 6.67, CI = 1.89, 23.52] was positively associated with method shifting.

**Conclusion:**

The proportion of method shifting from long to short term contraceptives was 48.5%. Women’s educational level, source of information, the aim behind using long acting contraceptives, and counseling adequacy were the main factors associated with method shifting. Therefore, health care providers better consider women’s educational level, provision of accurate information and adequate counseling are crucial in the provision of long acting contraceptive methods.

## Background

Family planning is the processes of having the amount of kids you want by employing various techniques to prevent unwanted or unplanned pregnancies. It is regarded as a component of every person’s or couple’s fundamental human rights [1, 2]. The rate of unintended pregnancy, early pregnancy, unsafe abortion, maternal morbidity, and death is reduced when contraceptive techniques are used properly [2, 3]. Contraceptive use generally enhances women’s health status by elevating their income, educational standing, and social connections [2, 3]. Poor contraceptive use can lead to unintended pregnancies, which can have detrimental effects on a woman’s health and wellbeing as well as the health of her family and the community as a whole [4, 5]. Infant, child, and maternal mortality can all be decreased by 40%, 10%, and 21%, respectively, with universal access to contraceptives [6].

Family planning was introduced globally in the 1960s as a sort of contemporary contraception to help women and couples achieve their desired fertility [7]. Male and female sterilization, the intrauterine device (IUD), implants, injectables, the pill, male and female condoms, emergency contraception, the standard day’s method (SDM), and the lactation amenorrhea technique (LAM) are all examples of contemporary contraceptive methods [8]. In addition, long-term methods of birth control are types of contemporary contraceptives, such as implants and intrauterine contraceptive devices (IUCD), which are practical and reliable ways to avoid unintended pregnancies. Since they are affordable, require few visits, and have a low risk of missed doses, they provide several benefits for consumers [9–11].

Due to the significant unmet demand for contraceptives in low resource nations, women there are exposed to a high rate of unwanted pregnancies [12]. Around the world, 64% of women of reproductive age who were married, in a union, or both used contraceptive methods, and roughly 33% of African women did as well [2]. 17% of women of reproductive age in Sub-Saharan African nations used modern methods of contraception [6]. The 2016 Ethiopian Demographic and Health Survey (EDHS) found that 35% of married or in-union women used contemporary contraception [8]. Additionally, according to the EDHS data, 29% and 71% of women who were married or in a union, respectively, used long- and short-term contraceptives [8].

Short acting contraceptives are quite effective at preventing unintended pregnancies. They must be used in short time intervals, ranging from single usage (such as the condom), daily ingestion (such as the pill), to up to three monthly applications, for them to operate best (e.g. injectable contraceptives) [13]. Users of these types of contraceptives must consider using or taking them frequently or each time they have intercourse. Additionally, each of these techniques can be applied repeatedly for many years. Because they are reversible, if users stop using them, the contraceptive effect quickly wears off, and women can become pregnant just as quickly as if they had taken no contraceptive at all [13].

Family planning was first practiced as a modern practice worldwide in the 1960s. Method shifting is the practice of switching from one group of contraceptives to another group, such as from long acting to short acting contraceptives or vice versa [14, 15]. When changing methods, women typically take some time (without using contraceptives) to determine which method to use, which exposes them to unwanted pregnancy. Additionally, some forms of contraception may be unfamiliar to women, and they may even require additional time to become used to it [9, 16–18]. Evidence indicated that long-acting contraceptive techniques are superior to short-acting ones in reducing the rate of unwanted pregnancy [14, 16, 19]. Additionally, compared to long-term contraceptive techniques, short-term contraceptive methods have a higher dropout rate. Additionally, due to a high failure rate or missed usage, the rate of unintended pregnancy and abortion is higher among short-term contraceptive users than it is among long-term users [5, 9, 18].

In Pakistan, IUCD users were the subject of a study, and the results revealed that 33.3% of the women switched to short-term methods [5]. In accordance with results of another study conducted in Malawi, 58% of women shifted to more effective ways, 24% to similar effective methods, and 18% to less successful methods [20]. According to a research conducted in Lusaka, Zambia, 13% of 145 implant users changed their methods, and of those, 89% switched to short-term family planning methods. Additionally, of the 40% of IUCD users, 33% changed their strategy; of these, 92% switched to short-term family planning techniques and 8% switched to Implanol [21]. A related study carried out in Kenya revealed that 31.3% of women switched from long-acting to short-acting contraceptives [22]. Besides, a study conducted in Honduras revealed that 14% of IUCD users shifted to other techniques [23]. Furthermore, a survey by Marie Stops in 2013 revealed that 25% of contraceptive users switched from short-term to long-term family planning methods [24].

Evidence from Ethiopia also shown that certain women are exposed to the country’s practice of switching from long to short term contraceptive techniques. According to a study done in Dire Dawa, 40.4% of women who use long-acting contraceptives changed their method of choice, with 29.8% switching to implants and 10.6% to IUCD [16]. Another study conducted in the Agarfa, Oromia region revealed that 4.2% of women switched from long-acting to short-acting contraceptives, while 3.4% switched from implants to injectables and 0.8% switched from implants to pills [11]. In the first year of using a contraceptive, 9.6% of users in Jimma Town, Southwest Ethiopia, switched to another technique [25]. Additionally, in the aforementioned study, the highest 12-month shifting rate was seen for tablets (19.9%), followed by condoms (12.4%), while implant (3.3%) and IUD rates were substantially lower (5.1%) [25].

By 2020, Ethiopia aimed to reach a Contraceptive Prevalence Rate (CPR) of 55% among women of reproductive age. Long-term reversible contraceptive techniques were intended to make up 48% of all methods, and they are crucial for achieving the national CPR goal because they are permanent. Each long-term form of birth control was intended to contribute as follows: IUD 15%, implant 33%, female sterilization 1.5%, and male sterilization 0.5% [26].

Even within the same nation, the proportion of women switching from long-term to short-term contraceptives varies greatly from study setting to study setting. Similar to this, there are regional differences in the components involved in method changing. The aforementioned evidence demonstrated the necessity of conducting the current investigation in that particular setting. Therefore, in Gondar City Administration, Northwest Ethiopia, this study was carried out to determine the percentage of method shifting from long-term to short-term contraceptive methods and its associated characteristics among reproductive age women.

## Methods

### Data source and study design

An institution based cross-sectional study was conducted from February to June 2018, at health facilities found under Gondar city administration. Gondar city is the capital city of north Gondar zone in Amara region, Northwest Ethiopia. The city is located 745kms far from Addis Ababa, the capital city of Ethiopia. The city is known as a world heritage cites with Faciledes castle and Epiphany is well known religious festival in the city. Gondar city has a total population of 327,661 of which 16, 3831 are females. The proportion of reproductive age women in the city was 23.9%. It has two public and one private, hospitals, 8 public health centers and 43 private clinics [27].

### Source population, sample size and sampling procedure

**Fig. 1:**
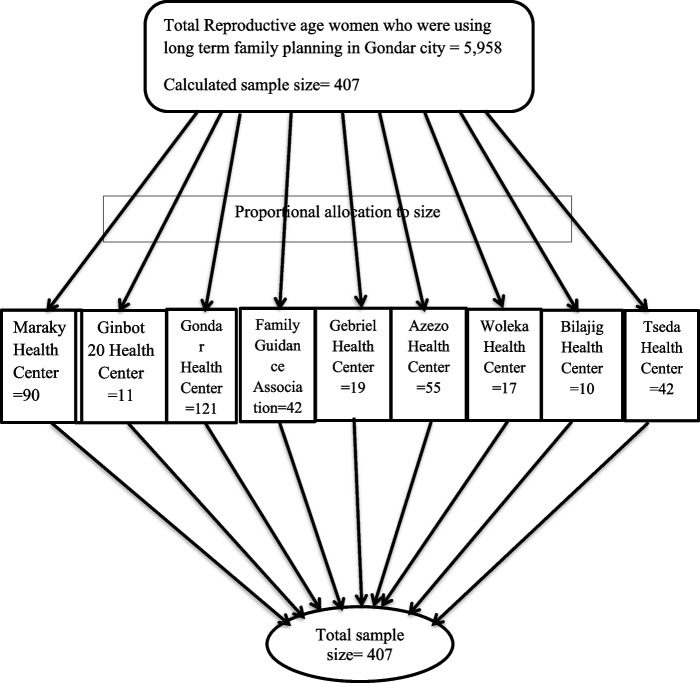
Schematic presentation showing the sampling procedure and proportional allocation of the calculated sample size for each health facilities included in the study, Gondar city

Women aged between 18 and 49 years, who had permanent resident in Gondar city and came for removal of long term contraceptives were included in the study. In addition, those women who shifted from long term to short term contraceptives were also included. Besides, those women who came for follow up visits on long term contraceptive methods were included Women who used the service at all governmental health centers and family guidance associations were included. Study subjects were selected using systematic random sampling technique and proportional to size was made for governmental health centers and Family Guidance Association (FGA) (Fig. 1). Women who were severely ill or unable to respond for the questionnaires were excluded from the study. Sample size was determined using single population proportion formula, considering the following assumptions: 40.4% as proportion(P) of woman who shift their method of choice from long to short term family planning [16], 95% confidence level and 5% degree of precision and 10% non-response rate. Finally, the sample size calculated for the study was 407.

### Variables and measurement

The outcome variable in this study, known as “method shifting”, was defined as the switch from the previously preferred long-term to short-term methods of contraception. That is, within three years of implant insertion, to any short-term approaches, and within five years of IUCD insertion, to any short-term methods [13]. Short-acting contraceptives are methods of birth control whose durations of action range from one day to three months and include injectables, pills, male and female condoms, emergency contraception, the standard day’s method (SDM), and the lactation amenorrhea method (LAM) [13]. Long-acting reversible contraceptive methods have durations of action that range from three to twelve years (intrauterine devices and implants). For women who didn’t have access to long-term contraceptive options and just a small number of short-term methods or one long-term technique were available at health facilities, a poor contraceptive method mix was operationalized. Additionally, the quality of counseling provided as part of family planning services was evaluated. Poor counseling was characterized as therapy that didn’t consider both the benefits and drawbacks of long-term family planning and didn’t provide enough time for it [28]. The effectiveness of the services provided by the providers was evaluated in light of the counseling, technique variety, and stock out information provided during family planning [28].

### Data collection tool and procedure

A pre-tested, structured, and interviewer-administered questionnaire that was first prepared in English and then translated in to Amharic (the local language) was used to collect the data. Five nurses with BSc degree were recruited as data collectors and a supervisor having a Master’s degree in public health specialty in reproductive health was recruited. Two days training was given to the data collectors and the supervisor about the objectives of the study, how to collect the data, and about the importance of keeping confidentiality of the responses of the respondents.

### Data processing and analysis

Data were entered into Epi-info version 7 software, and then it was exported to the Statistical Package for Social Sciences (SPSS) software version 20. The data were checked for completeness, cleaned, coded and for further analyzed using SPSS. Descriptive statistics were carried out for each of the variables and to ascertain the proportion of woman who shifted their method of choice. Then, findings were summarized and presented by frequency tables and charts. We employed the bivariable and multivariable logistic regression models to show the presence or absence of statistical association between independent variables with the outcome variable. The Hosmer and Lemshow goodness of fit test was used to select the best multivariable model and the *p*-value of the model fitness test was 0.830. In the bivariable logistic regression model, those factors having *p*-value of < 0.2 were fitted in to the multivariable model to adjust for the effect of potential confounding variables. Adjusted odds ratios with 95% confidence intervals (CI) and *P*-values were used to show the presence of association between independent and dependent variable (*P*-Value less than 0.05 were taken as statistically significant).

### Ethics approval

The University of Gondar Institutional Review Board (IRB) approved the study. Informed and written consent was obtained from all the respondents before the commencement of interviews with each respondent.

## Results

### Socio-demographic characteristics of the respondents

A total of 390 women participated in the study that made the response rate of the current study as 96%. The majority of the respondents, 315(80.8%), were urban residents. Concerning on the age of the respondents, the majority, 139(35.6%), ranged between18-24 years, with the mean age of 27 years having a standard deviation (SD) of ± 6.4. of the respondents were uneducated, while 133 (34.1%) had a monthly income of less than 1000 Ethiopian Birr (ETB) [19.3 USD] (Table 1).

**Table 1:** Socio-demographic characteristics of women of reproductive age (18–49 years) in Health facilities of Gondar City, 2018

**Variables**	**Frequency**	**Percent**
**Age of the women (in year)**		
18–24	139	35.6
25–29	138	35.4
30–34	51	13.1
Above 35	62	15.9
Mean ± SD	27 ± 6.4	----
**Place of residence**		
Urban	315	80.8
Rural	75	19.2
**Educational status**		
Uneducated	148	37.9
Elementary (1–8)	102	26.2
Secondary (9–12)	98	25.1
Diploma and above	42	10.8
**Marital status**		
Married	305	78.2
Divorced	23	5.9
Widowed	11	2.7
Single	51	13.2
**Religion**		
Orthodox Christian	298	76.4
Muslim	82	21.1
Others^a^	10	2.5
**Ethnicity**		
Amhara	299	76.7
Oromo	91	23.3
**Employment status**		
Student	77	19.7
Merchant	53	14.1
Daily laborer	15	3.5
House wife	173	44.3
Government employee	50	12.7
Farmer	22	5.7

^a^Protestant, catholic

### Reproductive history of the women

More than half, 234(60%), of women started first sexual intercourse at age before 18 (mean age 17.9, SD ± 2.8) and about 186(53.4%) of women married before the age of 18 years. In addition, nearly half of the respondents, 139(48.4%), had history 1–2 pregnancies. Moreover, one-fifth, 72(23.5%), of the women had history of home delivery. Nearly half, 147(47.9%) of the women attended antenatal care at least once (Table 2).

**Table 2:** Reproductive history of women of reproductive age (18–49 years) who uses health facilities in Gondar city, 2018

Variables	Frequency	Percent
**Age at first sexual intercourse (in years)**		
Less than 18	234	60
19–24	150	38.5
Above 25	6	1.5
**Age at 1st marriage**		
Less than 18	186	53.4
19–24	147	42.3
Above 25	15	4.3
**Number of pregnancy**		
1–2	144	48.5
3–4	113	37.6
Above 5	50	13.9
**Place of delivery**		
Health center	87	28.4
Hospital	123	40.2
Home	72	23.5
Home delivery assisted by traditional birth attendants	24	7.8
**Ideal Number of Children**		
1–2	246	63.6
3–4	110	28.7
Above 5	30	7.8
**Number of ANC visit (** ***n*** ** = 307)**		
No	131	42.7
1–2	147	47.9
3–4	29	9.4

### Method shifting from long to short term contraceptive methods

Nearly half, 113(48.5%) (With 95% CI: 43.8–53.3), of respondents had shifted from long term to short term contraceptive methods. More than a third, 76(37.1%), shifted their previous method between 6 and 12 months. Method shifting from implant to other short term contraceptive methods was (34.0%) and shifting from IUCD was (14.5%). More than one-fifth (25.9%) of women shifted the contraceptive within the first six months of insertion (Fig. 1).

**Fig. 2:**
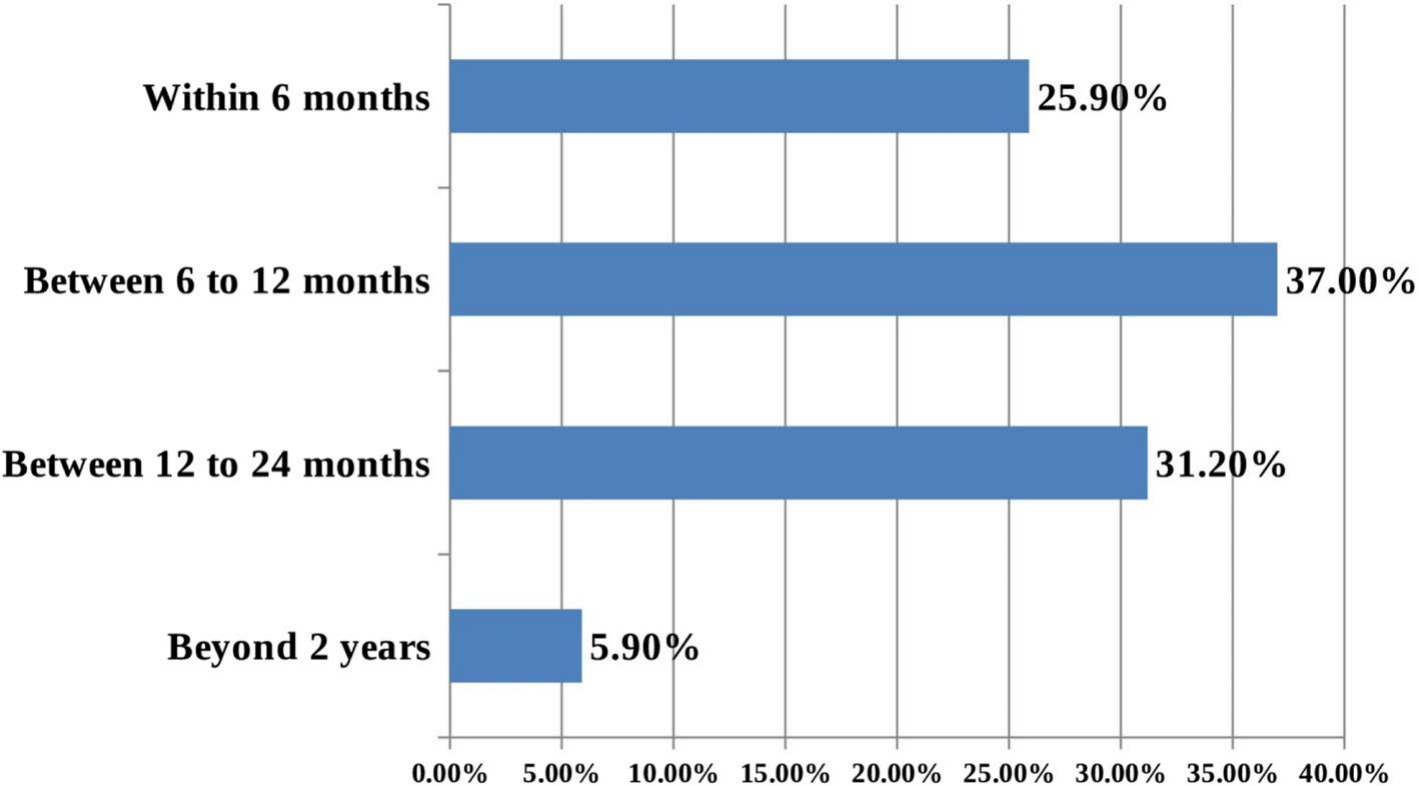
Showing time of method shifting from long to short term contraceptives

**Fig. 3:**
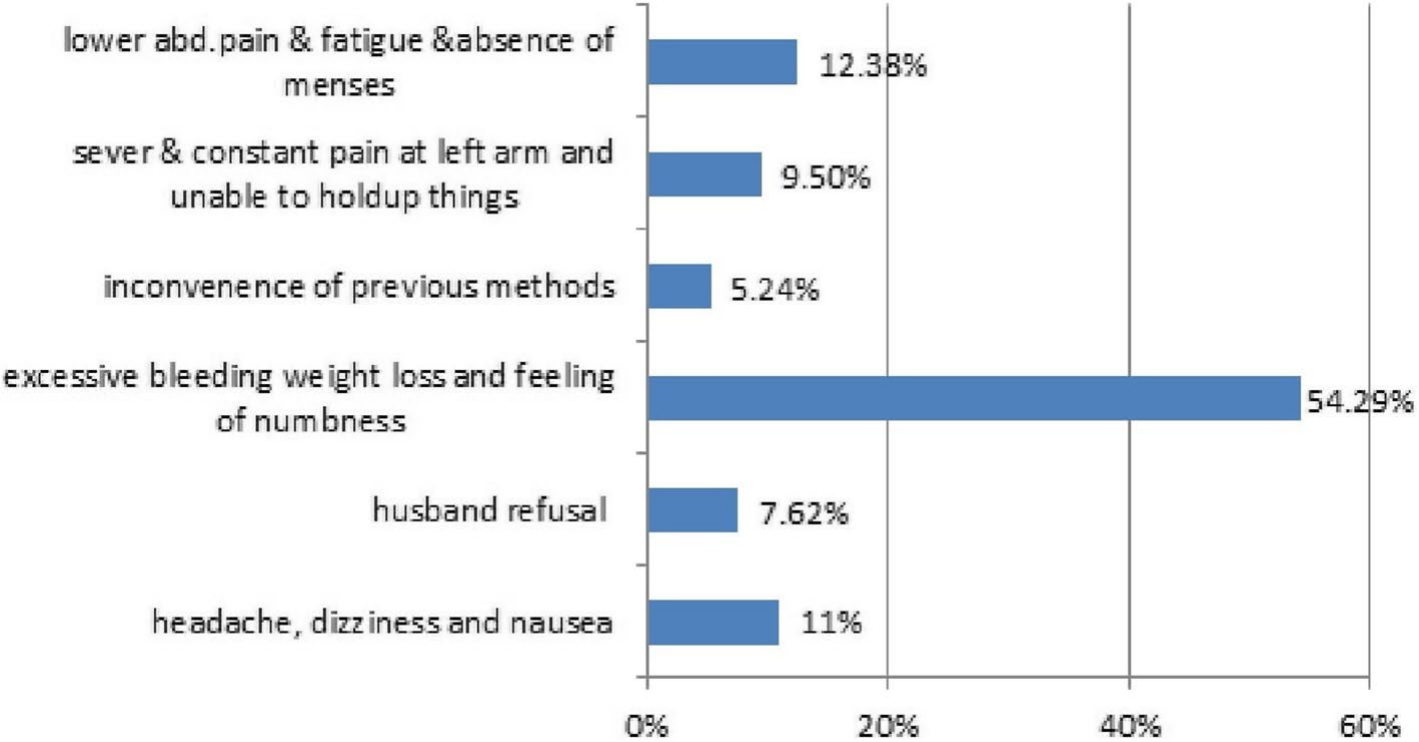
Showing the main reasons of method shifting from long to short term contraceptives [29]

About 44(11%) of women reported that they were pregnant while using modern contraceptive methods such as Depo-Provera (DEPO), 23(5.9%), and pills, 15(3.8%).The main reasons for method shifting were side effects 176 (87.14%) such as bleeding, weight loss and feeling of numbness (Figs. 2 and 3).

Regarding the source of information, more than half, 222 (56.9%), of them get information about contraceptive methods from health care providers. Moreover, about 14.4% of them revealed that counseling service provided at health institution was not adequate (Table 3).

**Table 3:** Contraceptive history of women of reproductive age (18–49 year) who were using health facilities in Gondar city, northwest Ethiopia, 2018

Variables	Frequency	Percent
**Reason of family planning use**		
Birth spacing	230	59
Birth limiting	160	41
**Over all method shifting**		
Yes	232	59.5
No	158	40.5
**Method shifting from long to short term contraceptives**		
Yes	113	48.5
No	119	52.5
**Pregnancy while using family planning method**		
Yes	44	11
No	346	89
**Source of information for long acting contraceptive**		
Health facility	233	59.7
Colleague	77	19.7
Media	71	18.2
Others	9	2.4
**Counseling service at the health facility**		
Satisfied	334	85.6
Not-satisfied	56	14.4

### Factors associated with method shifting from long to short term contraceptive methods

Two models called the bivariable and multivariable logistic regression analysis were carried out to identify factors associated with the outcome variable. In the first model (the bi-variable logistic regression analysis), age, educational status, counseling about long acting contraceptives during ANC visit, reason for family planning use, previous number of children, demand of children, counseling satisfaction during family planning service, marital status, experience of pregnancy while using modern family planning, information about long term family planning were eligible factors found to have significant association(having *p*-value of less than 0.2) with method shifting from long to short term contraceptive methods. All of those variables having *P*-value of < 0.2 in the bivariable analysis were fitted in to the second model (the multivariable logistic regression model). While in multivariable logistic regression analysis, educational status (had secondary education), reason for contraceptive use (for limiting) and had providers counseling about long term family planning during ANC visits were found to be negatively associated with method shifting, on the other hand, source of information about long term contraceptive methods (from colleague) was positively associated with method shifting from long to short term contraceptive methods having *p*-value of less than 0.05.

When compared to women with no education, women with a secondary education had an 83% lower chance of switching from long-term to short-term contraceptive methods [AOR = 0.17, CI = 0.06, 0.45].

Women who used long-acting contraceptives for limiting purposes were 75% lower odds to switch from them to short-acting methods than women who used long-acting contraceptives for spacing [AOR = 0.25, CI=[0.11,0.56].

The odds of method shifting from long to short term contraceptive methods was 6.67 times more likely to occur among women who get information about long term contraceptive methods from colleague as compared to those who get information about long term contraceptives from health providers [AOR = 6.67, CI = 1.92, 23.02].

Additionally, when compared to women who received insufficient long-acting contraceptive counseling during ANC visits, the odds of switching from a long-acting method to a short-acting method were 78% lower in the former group [AOR = 0.22, CI = 0.09,0.51] (Table 4).

**Table 4:** Factors associated with method shifting from long acting family planning method among women of reproductive age (18–49 years) in Gondar city, northwest Ethiopia, 2018

Variables	Method shifting		Crude Odds Ratio	Adjusted Odds Ratio	*P*-Value
	Yes N (%)	No N (%)	[95% CI]	[95% CI]	
**Age of women (in years)**					
Less than 24	72 (35.8%)	76 (40.2%)	1	1	
25–29	63 (31.3%)	71 (37.6%)	1.07 (0.67,1.71)	1.82 (0.71,4.65)	
30–34	28 (13.9%)	19 (10.1%)	0.64 (0.33,1.25)	1.13 (0.3, 4.06)	
Above 35	38 (18.9%)	23 (12.2%)	0.57 (0.31,1.06)	0.57 (0.31, 1.06)	0.20
**Educational status**					
Uneducated	69 (34.3%)	79 (41.8%)	1	1	
Elementary	42 (20.9%)	60 (31.7%)	1.25 (0.75, 2.08)	1.06 (0.35,6.69)	
Secondary	65 (32.3%)	33 (17.5%)	0.44 (0.26,0.75)	0.18 (0.07,0.51)*	0.01
Diploma and above	25 (12.4%)	17 (9%)	0.59 (0.30,1.19)	0.95 (0.25,3.57)	
**Marital status**					
Married	163 (81.1)	143 (75.7)	1	1	
Single	25 (12.4)	20 (10.6)	0.912 (0.49,1.71)	1.878 (0.12,29.37)	
Others	13 (6.5)	26 (13.8)	2.280 (1.13,4.60)	3.704 (0.60,23.02)	0.41
**Number of pregnancy**					
1–2	49 (33.3%)	76 (54.3%)	1	1	
3–4	75 (51%)	50 (35.7%)	0.43 (0.26,0.71)	0.32 (0.13,0.75)	
Above 5	23 (15.6%)	14 (10%)	0.39 (0.18,0.84)	0.25 (0.06,1.12)	0.09
**Number of children**					
1	53 (35.8)	57 (40.1%)	1	1	
2–4	79 (53.4%)	82 (57.7%)	0.97 (0.59,1.57)	0.82 (0.34,1.94)	
Above 5	16 (10.8%)	3 (2.1%)	0.17 (0.05,0.63)	0.12 (0.01,0.96)	0.25
**Number of children to have**					
1–2	113 (56.8%)	133 (70.7%)	1	1	
3–4	67 (33.7%)	44 (23.4%)	0.56 (0.35,0.88)	0.85 (0.36,2.02)	
Above 5	19 (9.5%)	11 (5.9%)	0.49 (0.23,1.08)	0.77 (0.18,3.27)	0.16
**Have you experience pregnancy while using on Family planning**					
Yes	16 (8.0%)	28 (14.8%)	2.01 (1.05,3.85)	4.31 (0.74,25.26)	0.11
No	185 (92%)	161 (85.2%)	1	1	
**Got enough counseling on long term contraceptive during ANC visit**					
Yes	44 (49.4%)	45 (50.6%)	0.35 (0.18,0.65)	0.20 (0.08,0.50)*	0.01
No	23 (25.3)	68 (74.8%)	1	1	
**Have you satisfied on Adequate counseling during family planning**					
Yes	178 (88.6%)	156 (82.5%)	0.61 (0.34,1.09)	0.59 (0.18,1.88)	0.51
No	23 (11.4%)	33 (17.5%)	1	1	
**Reasons for family planning use**					
Birth limiting	95 (47.3%)	65 (34.4%)	0.59 (0.39,0.88)	0.26 (0.11,0.60)*	0.001
Pregnancy spacing	106 (52.7%)	124 (65.6%)	1	1	
**Source of information about long term family planning**					
Health facility	142 (70.6%)	91 (48.1%)	1	1	
Colleague	39 (19.4%)	38 (20.1%)	2.28 (1.28,4.06)	6.67 (1.89,23.52)*	< 0.001
Media /others	20 (10%)	60 (31.8%)	2.16 (1.04,4.47)	2.31 (0.51,9.76)	

*Found statistical significant at *p*-value ≤ 0.05

## Discussion

This study evaluated the proportion and factors related to method switching from long to short term contraceptive methods among reproductive age women (18–49 years) in Gondar city administration, northwest Ethiopia,

In this study the overall proportion of method shifting from long to short acting contraceptive methods was 48.5% [CI = 43.8,53.3]. The finding was in line with the study conducted in Egypt 46% [30].

The proportion of method shifting from long to short acting contraceptive method in this study was lower as compared with those studies conducted in Vietnam 80% [31] Malawian 58% [20], Zambia 89% [21], and Vietnam 66.7% [32]. The possible justification for the lower proportion of method shifting from long to short term contraceptives in the current study as compared to those of the above studies might be due to the fact that Ethiopian government has given more emphasis for long acting family planning method use, through nurses, midwives and health extension programs even at lower level of the health care system (primary health care unit) [33]. Moreover, variations in study participants among studies might be the possible justification for the differences. For instance, only post-partum women were included in Malawi and HIV concordant positive or discordant couples were study participants included in Zambia.

In contrast to our findings, method shifting from long to short term contraceptive methods was relatively lower in studies conducted in Kenya (31.3%) [22], Pakistan (33.3%) [5], Agarfa, Oromia region (4.2%) [11], Dire Dawa (40.4%) [16]. This might be due to differences in socio-demographic characteristics of participants. For instance, 91% of participants in the study conducted in Dire Dawa were married, while 76% of women in the current study were single. As a result, married women might be less likely shift their method of choice from long to short term contraceptive methods as compared to single women.

According to the multi-variable logistic regression analysis, the odds of method shifting from long to short acting contraceptive methods among women who had secondary education was 83% less likely when compared with uneducated women [AOR = 0.17, CI = 0.06,0.45]. This finding was consistent with other studies done in Dire Dawa and Senegal [16, 31, 34]. This might be due to the fact that women who were educated get information on long term family planning methods.

The odds of method shifting from long to short term family planning methods were 75% less likely among women who used contraceptives for limiting purpose as compared to women who used family planning for spacing [AOR = 0.25, CI = 0.11,0.56]. This finding was consistent with those studies done in Dire Dawa, Jimma and Hosanna [16, 25, 35]. This might be due to the fact that women who use long term contraceptives for limiting purpose may be those who reached their desired family size so they decide to have no more children for future they were used long term contraceptives instead of shifting to short term family planning methods.

The odds of method shifting from long to short term contraceptive methods among women who get information about long term family planning from colleague were 6.65 times more likely to occur as compared to women who get information from health care providers [AOR = 6.67, CI = 1.92,23.02]. This finding was consistent with those studies done in Dire Dawa [16]. Colleague information may propagate a range of long-term contraceptive myths, misunderstandings, and false notions that expose women to technique shift.

The odds of method shifting from long to short term contraceptive methods were 78% less likely among women who get counseling about long term family planning during ANC follow up compared to those who didn’t get counseling [AOR = 0.22, CI = 0.09,0.51]. This finding was consistent with the study conducted in Jimma town, Ethiopia [25]. This might be due to the fact that women who attend all ANC follow up will get counseling about long term contraceptives during the course of ANC follow-up to get adequate information about long term contraceptive methods.

The study’s findings have some programmatic and policy implications in that it’s crucial to reinforce the program while designing long-acting contraceptives by taking the stated factors into account. The results also have some practical implications in that it is vital to provide intensive counseling while providing long-acting contraceptives, taking into account women’s educational position, and to provide adequate information in order to minimize method shift.

### Limitations of the study

Since we employed cross-sectional study design, the identified factors cannot be considered as the real causes for method shifting.

## Conclusion

The proportion of method shifting from long to short term contraceptives was 48.5%. Women’s educational level, source of information, the aim behind using long acting contraceptives, and counseling adequacy were the main factors associated with method shifting. Therefore, health care providers better to consider women’s educational level, provision of accurate information and adequate counseling are crucial in the provision of long acting contraceptive methods.

## Data Availability

All the data required to make the conclusions of the results of this study are included in the manuscript.

## Abbreviations

AOR: Adjusted Odds Ratio
CI: Confidence Interval
COR: Crude Odds Ratio
CPR: Contraceptive Prevalence Rate
DEPO: Depo-Provera
EDHS: Ethiopian Demographic Health Survey
FGA: Family Guidance Association
IMP: Implant
IUCD: Intra Uterine Contraceptive Device
SPSS: Statistical Package for Social Sciences
